# Emergence of *fexA* in Mediating Resistance to Florfenicols in Campylobacter

**DOI:** 10.1128/AAC.00260-20

**Published:** 2020-06-23

**Authors:** Biao Tang, Yizhi Tang, Ling Zhang, Xiao Liu, Jiang Chang, Xiaodong Xia, Hua Yang, Zhangqi Shen

**Affiliations:** a State Key Laboratory for Managing Biotic and Chemical Threats to the Quality and Safety of Agro-products, Zhejiang Academy of Agricultural Sciences, Hangzhou, China; b Institute of Quality and Standard for Agro-products, Zhejiang Academy of Agricultural Sciences, Hangzhou, China; c Key Laboratory of Bio-Resource and Eco-Environment of Ministry of Education, Animal Disease Prevention, Sichuan University, Chengdu, China; d Food Safety Key Laboratory of Sichuan Province, College of Life Sciences, Sichuan University, Chengdu, China; e College of Food Science and Engineering, Northwest Agriculture and Forestry University, Yangling, China; f Beijing Key Laboratory of Detection Technology for Animal-Derived Food Safety, Beijing Laboratory of Food Quality and Safety, College of Veterinary Medicine, China Agricultural University, Beijing, China

**Keywords:** *Campylobacter*, *fexA*, multidrug resistance, food safety

## Abstract

Florfenicol belongs to a class of phenicol antimicrobials widely used as feed additives and for the treatment of respiratory infections. In recent years, increasing resistance to florfenicol has been reported in *Campylobacter* spp., the leading foodborne enteric pathogens causing diarrheal diseases worldwide. Here, we reported the identification of *fexA*, a novel mobile florfenicol resistance gene in *Campylobacter*. Of the 100 Campylobacter jejuni strains isolated from poultry in Zhejiang, China, 9 were shown to be *fexA* positive, and their whole-genome sequences were further determined by integration of Illumina short-read and MinION long-read sequencing. The *fexA* gene was found in the plasmid of one strain and chromosomes of eight strains, and its location was verified by S1 nuclease pulsed-field gel electrophoresis (S1-PFGE) and Southern blotting. Based on comparative analysis, the *fexA* gene was located within a region with the *tet*(L)-*fexA-catA-tet*(O) gene arrangement, demonstrated to be successfully transferable among C. jejuni strains. Functional cloning indicated that acquisition of the single *fexA* gene significantly increased resistance to florfenicol, whereas its inactivation resulted in increased susceptibility to florfenicol in *Campylobacter*. Taken together, these results indicated that the emerging *fexA* resistance is horizontally transferable, which might greatly facilitate the adaptation of *Campylobacter* in food production environments where florfenicols are frequently used.

## Abstract (precis)

Florfenicol belongs to a class of phenicol antimicrobials widely used as feed additives and for the treatment of respiratory infections. In recent years, increasing resistance to florfenicol has been reported in *Campylobacter* spp., the leading foodborne enteric pathogens causing diarrheal diseases worldwide. Here, we reported the identification of *fexA*, a novel mobile florfenicol resistance gene in *Campylobacter*. Of the 100 Campylobacter jejuni strains isolated from poultry in Zhejiang, China, 9 were shown to be *fexA* positive, and their whole-genome sequences were further determined by integration of Illumina short-read and MinION long-read sequencing.

## INTRODUCTION

Florfenicol is a fluorinated thiamphenicol derivative belonging to the broad-spectrum antimicrobial agents exclusively approved for use in veterinary medicine (1). It has been licensed in China for the control of respiratory tract diseases and enteric infections in food-producing animals since 1999 (2). However, the excessive use of florfenicol as an antimicrobial chemotherapeutic agent has resulted in bacterial species acquiring resistance to this agent. Several phenicol resistance-specific genes have been reported for florfenicol-resistant bacteria of animal and human origin, including the *fexA*, *fexB*, *pexA*, and *floR* phenicol-specific exporter genes, *optrA*, a ribosomal protection protein gene, the gene encoding RE-CmeABC, a functionally enhanced multidrug efflux pump variant (3–5), and *cfr*, the multidrug resistance gene encoding a 23S rRNA methyltransferase that confers resistance to phenicols, as well as 4 other structurally unrelated antimicrobial agents (lincosamides, oxazolidinones, pleuromutilins, and streptogramin A) (6).

*Campylobacter* is the leading cause of bacterial foodborne illnesses worldwide. According to data from the World Health Organization, the estimated incidence of gastroenteritis due to infections by *Campylobacter* spp. in high-income countries is between 4.4 and 9.3 per 1,000 people (7). Contaminated undercooked poultry meat is the main source of infection for human campylobacteriosis, with ruminant *Campylobacter* also being a significant contributor to foodborne illnesses. Numerous reports have indicated that *Campylobacter* has become increasingly resistant to antimicrobial agents used in animals and clinical settings (8–10). As a foodborne pathogen transmitted via foodborne routes, *Campylobacter* is constantly exposed to antimicrobial agents used for food production. Hence, in dealing with antimicrobial selection, *Campylobacter* has evolved various mechanisms of resistance to such antimicrobials. Some of the mechanisms have been shown to confer resistance to a specific class of antimicrobials, whereas others might confer multidrug resistance (11). In addition to the previously characterized intrinsic mechanisms in mediating antibiotic resistance, several new antibiotic resistance mechanisms have emerged in *Campylobacter* in recent years. These mechanisms include the Erm(B) rRNA methylase, mediating macrolide resistance (12–14), RE-CmeABC, a functionally enhanced multidrug efflux pump variant (5), a novel *fosX*^CC^ gene conferring fosfomycin resistance (15), and the Cfr(C) rRNA methyltransferase, mediating multidrug resistance (16, 17). It should be noted that the presence of RE-*cmeABC* not only confers resistance to florfenicol but also contributes to the increased resistance to clinically important antimicrobial agents, such as ciprofloxacin and erythromycin (5). In addition, multiple characterized resistance mechanisms could form multidrug resistance genomic islands (MDRGIs), which could confer a fitness advantage under various antimicrobial selections in *Campylobacter* (10, 12, 17, 18).

During our routine surveillance, we noticed that several *Campylobacter* isolates showed extremely high levels of resistance to florfenicol. In this study, we aimed to identify and characterize novel florfenicol resistance mechanisms in *Campylobacter*. A natural transformation assay indicated that florfenicol resistance could be transferred among *Campylobacter* organisms. Whole-genome sequencing of transformants revealed the presence of the previously uncharacterized *fexA* florfenicol resistance gene in *Campylobacter* isolated from poultry. We further analyzed the function and genetic environments of *fexA*.

## RESULTS

### Identification of *fexA* associated with resistance to florfenicol.

Between March 2018 and January 2019, a total of 100 Campylobacter jejuni strains were isolated from 3 poultry farms, 2 slaughterhouses, and 6 supermarkets in Zhejiang Province, China. During our surveillance study regarding *Campylobacter* from poultry, we noticed that 7 of the C. jejuni isolates exhibited a florfenicol MIC value of ≥64 mg/liter, which was higher than what was previously reported for *Campylobacter*. To search for the mechanisms mediating high resistance to florfenicol, we performed PCR analysis of the previously characterized florfenicol resistance genes in *Campylobacter*, namely, *cfr*(C) and RE-*cmeABC*. However, these 2 genes were absent in some of our florfenicol-resistant isolates, indicating the presence of a potentially novel mechanism conferring resistance to florfenicol. In addition, natural transformation assays revealed that florfenicol resistance in C. jejuni ZS005 was transferable to C. jejuni NCTC 11168. Specifically, the MIC value for florfenicol was shown to be increased 32-fold in the ZS005NT11168 transformant compared with that of the parent, C. jejuni NCTC 11168 (Table 1). This finding indicated that the performed transformation resulted in the transfer of florfenicol resistance mechanisms from C. jejuni ZS005 to ZS005NT11168. To examine this possibility, we performed whole-genome sequence analysis of ZS005NT11168. Comparative analysis of the draft genome of ZS005NT11168 with the complete genome of NCTC 11168 (accession no. NC_002163) revealed the presence of a 9,982-bp segment containing a 1,428-bp gene with 99.51% (7 single nucleotide polymorphisms [SNPs]) similarity to the *fexA* gene in Staphylococcus lentus (19), which could encode a phenicol-specific efflux pump conferring resistance to florfenicol (see Fig. S1 in the supplemental material). Beyond this replacement, no other insertions/deletions or plausible SNPs were observed in ZS005NT11168. Further PCR amplification screening indicated that all the 7 C. jejuni strains mentioned above are positive for *fexA*. In addition, the presence of *fexA* was identified in another 2 C. jejuni strains with a florfenicol MIC value of 32 mg/liter. These results indicated that the natural transformation led to the allelic exchange of *fexA*-containing multidrug resistance genomic islands (MDRGIs) between the donor and recipient strains, suggesting that the acquisition of the *fexA* gene from ZS005 might contribute to the elevated resistance to florfenicol in ZS005NT11168.

**TABLE 1:** Key bacterial strains used in this study and MIC values of florfenicol for various C. jejuni strains as determined by the broth dilution method

Bacterial strain	Description or relevant genotype	MIC (mg/liter)^*a*^
NCTC 11168	Wild-type C. jejuni lacking *fexA*	1
ZS005NT11168	NCTC 11168 derivative transformants	32 (↑32)
11168*+fexA*	NCTC 11168 derivative; *rrs*::*fexA*	32 (↑32)
ZS005	C. jejuni isolate containing *fexA*	64
ZS005 Δ*fexA*	ZS005 derivative; Δ*fexA*::Kan^r^	0.5 (↓128)
ZS006	C. jejuni isolate containing *fexA*	64
ZS006 Δ*fexA*	ZS005 derivative; Δ*fexA*::Kan^r^	0.5 (↓128)

a Numbers in parentheses indicate fold changes over the wild-type control, either increased (↑) or decreased (↓).

### Functional confirmation of *fexA*.

To determine the role of *fexA* in florfenicol resistance in C. jejuni, insertional mutagenesis was used to construct isogenic mutations in ZS005 and ZS006. The generated *fexA* mutants were compared with the parent strains for susceptibility to florfenicol compounds using the broth dilution method. According to MIC results obtained from the broth dilution method, inactivation of *fexA* in these isolates resulted in 64- and 128-fold reductions in their MICs for florfenicol, respectively (Table 1).

To further confirm the function of *fexA*, its encoding sequence along with the promoter region was inserted into C. jejuni NCTC 11168 between the 16S and 23S rRNA genes. The sequences of promoter regions from eight chromosomes are consistent (Fig. S2). According to the MIC results, acquisition of the single *fexA* gene in NCTC 11168 resulted in a 32-fold increase in its MIC for florfenicol (Table 1) but had no effects on the MICs for other tested antimicrobial agents (data not shown), indicating that *fexA* specifically contributes to the resistance to florfenicol. Collectively, these results clearly demonstrated the specific role of *fexA* in the florfenicol resistance phenotype in *Campylobacter*.

### Genetic environments of *fexA*.

To identify the associated genetic environments of *fexA*, the whole genomes of 9 *fexA*-containing C. jejuni isolates were sequenced. At least 100× coverage of raw reads from Illumina sequencing were obtained for each isolate. Draft genomes were *de novo* assembled using the CLC Genomics Workbench (version 8.5). The number of contigs ranged from 51 to 232, while the *N*_50_ of contigs ranged from 43 to 521 kb for the isolates assembled by the CLC Genomics Workbench. Due to the short reads generated by Illumina sequencing and the high number of insertion elements, the assembled *fexA*-carrying contigs for the 9 isolates were relatively short, ranging from 2.3 to 3.8 kb.

In order to obtain the genetic environments of *fexA*, these 9 isolates were resequenced by MinION long-read sequencing to generate complete chromosomes and plasmids. Draft genomes of these isolates were 1,612,562 to ∼1,828,288 bp in length, with a GC content of 30.15 to ∼30.64%.

**FIG 1:**
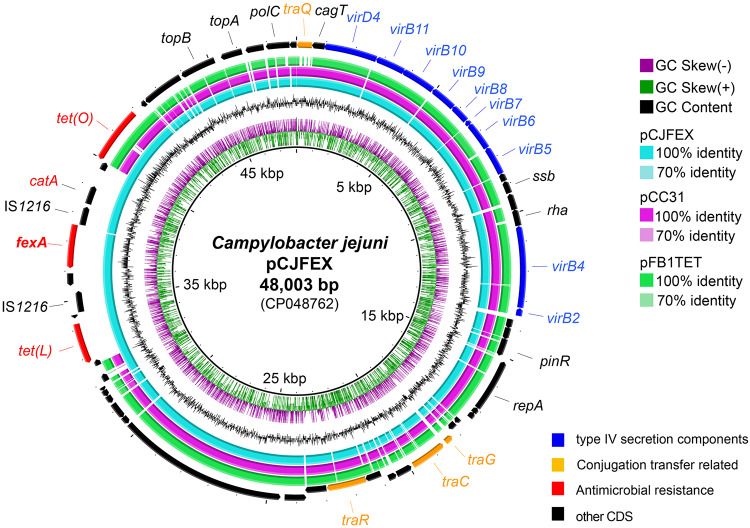
Alignment of the *fexA*-bearing pCJFEX plasmid from the ZS004 C. jejuni strain against homologous plasmids. pCJFEX, labeled in cyan, was aligned to the pCC31 and pFB1TET plasmids using BLAST Ring Image Generator (BRIG) software. The *tet*(L)-*fexA-catA-tet*(O) gene arrangement in pCJFEX represents an insertion sequence compared with the other two plasmids.

Comparative analysis of complete genomes revealed that the ZS004 C. jejuni strain had a 48,003-bp *fexA*-carrying pCJFEX plasmid (Fig. 1). Note that pCJFEX contains 58 predicted open reading frames (ORF) with a 29.98% GC content. In addition, this plasmid from the ZS004 strain can be aligned very well to pCC31 (GenBank accession no. AY394560) and pFB1TET (GenBank accession no. CP011017) (99% in both identity and coverage) recovered from Campylobacter coli, with a conserved backbone associated with the plasmid transfer by conjugation.

**FIG 2:**
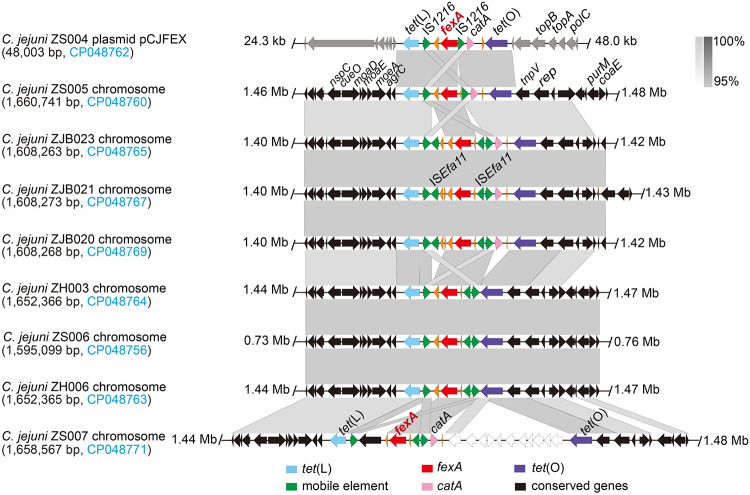
Genetic environment of *fexA* in plasmid or genomes of C. jejuni isolates and comparison of the *fexA*-carrying regions. Arrows indicate the direction of transcription of the genes. Regions of >95% homology are marked by gray shading. Genes are differentiated by color. Brightly colored ORF represent the *tet*(L)-*fexA-catA-tet*(O) gene arrangement. These multidrug resistance genomic islands (MDRGIs) could be divided into 5 different types. ZJB023, ZJ021, and ZJ020 carry the same MDRGIs, whereas ZH003, ZS006, and ZH006 belong to a group containing the same MDRGIs.

The *fexA* gene on the plasmid was demonstrated to be located within a 9,982-bp region, with a *tet*(L)-*fexA-catA-tet*(O) gene arrangement. The 9,982-bp region had 2 intact copies of IS*1216* in the same orientation and contained 5 genes, including *tet*(L) and *tet*(O) for tetracycline resistance, *fexA* for florfenicol resistance, *catA* for phenicol resistance, and a gene encoding a hypothetical protein. The *tet*(L)-*fexA-catA-tet*(O) gene arrangement in pCJFEX represented an insertion sequence (IS) compared with the other 2 plasmids. Similar regions with ≥95% nucleotide sequence identity were also identified in the chromosomes of 8 other C. jejuni isolates (ZH003, ZH006, ZS005, ZS006, ZS007, ZJB020, ZJB021, and ZJB023) (Fig. 2). All these MDRGIs were shown to be located between the *agrC* and *repA* genes. Based on colinear alignment, these regions could be divided into at least 4 kinds of sequences according to the number of mobile elements inserted (Fig. 2). The presence of the IS element indicated the potential translocation of the *fexA*-carrying region between different plasmids and integration into the chromosome. Further, we found that the IS*1216* around *fexA* and the IS*1216* copies played the key role in integrating this *fexA*-carrying segment based on retrospective analysis (Fig. S3).

### Molecular typing and phylogenetic analysis of the *fexA*-carrying C. jejuni isolates.

**FIG 3:**
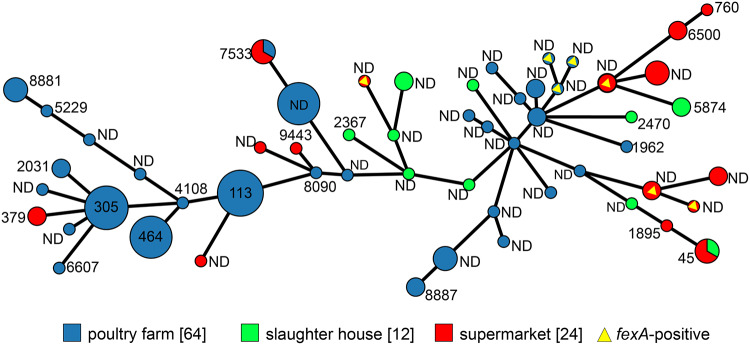
Minimum spanning tree of *fexA*-positive and *fexA*-negative C. jejuni isolates according to MLST and gene allele proﬁle. The tree was created by BioNumerics (Applied Maths, Belgium). Each node within the tree represents a single ST. The size of the nodes is proportional to the number of isolates represented by said node. Selected nodes are labeled with corresponding ST, phylogenetic group, and number of represented isolates. ND, unknown ST.

The combined data indicated the significant horizontal dissemination of the *fexA* gene through C. jejuni isolates of different origins (Fig. 3). Of 100 Campylobacter jejuni isolates, 52 were identified as known sequence types (STs), including 21 STs. Among them, ST113, ST305, and ST464 were shown to include a large number of isolates. In contrast, 48 strains did not match the known ST, including the 9 *fexA*-positive strains. These 9 strains were shown to belong to 7 different unknown STs, 3 on poultry farms and 4 in supermarkets, meaning that these 9 *fexA*-positive isolates were multisourced (Fig. 3).

## DISCUSSION

In this study, we identified the *fexA* gene as an emerging mechanism in mediating resistance to florfenicol in C. jejuni. This finding was supported by multiple pieces of evidence. The presence of *fexA* was associated with elevated MIC values for florfenicol (Table 1), whereas its inactivation resulted in a 128-fold reduction in the MIC value. Concomitantly, cloning of *fexA* into a C. jejuni strain lacking *fexA* resulted in the recipient strain exhibiting a 32-fold increase in its MIC for florfenicol, whereas no effect was observed on the MIC values for other tested antimicrobial agents. These results indicated that *fexA* could function as a florfenicol-specific resistance mechanism. Importantly, this finding further enriched our knowledge of the various mechanisms of florfenicol resistance in *Campylobacter*.

Due to the use of florfenicol as well as other antimicrobial agents in poultry production, *Campylobacter* spp. in poultry must be able to deal with the toxicity and selective pressure deriving from these compounds. Accumulated studies have indicated that *Campylobacter* isolates of animal origin have shown increased resistance to various antimicrobial agents, with continuous acquisition of different mechanisms of resistance to such compounds (5, 11). In recent years, we have characterized different multidrug resistance genomic islands (MDRGIs), which confer resistance to macrolides, aminoglycosides, tetracyclines, fosfomycin, and so on (10, 12–15, 18). Those genomic islands showed different GC contents when compared with the whole genome of *Campylobacter*, suggesting that these contents originated from different species. In this study, the characterized *fexA* gene was shown to be associated with *tet*(L), *tet*(O), and *catA*, forming a novel multidrug resistance genomic island. Although a Basic Local Alignment Search Tool (BLAST) search against the GenBank database could not match the exact same MDRGI from the other species, similar MDRGIs containing *fexA* associated with IS*1216* were found in *Enterococcaceae* (Fig. S3). The GC content for this MDRGIs was reported to be 36.4%, which was different from the GC content of the *Campylobacter* genome (∼30%), suggesting that *Campylobacter* might have obtained this MDRGI from other species. Interestingly, whole-genome analysis of the *fexA*-harboring isolates indicated that they were generally diverse, with *fexA* being either in the chromosome or on the plasmid, suggesting that *fexA* could spread by horizontal gene transfer. Accordingly, we demonstrated the transferability of this MDRGI in the laboratory. Moreover, searching the *fexA* gene in the GenBank database using BLAST revealed that one C. coli isolate from PulseNet in the United States contained the *fexA* gene; however, we could not obtain the genetic environment of the *fexA* gene, probably due to the short read produced by the second-generation sequencing method. During our preparation of the manuscript, the whole-genome sequence of another *fexA*-carrying C. coli strain, 16SHKX65C (GenBank accession no. CP038868), was released from China (Fig. S3). Given that *Campylobacter* is known to be naturally transformable, it is expected that the prevalence of *fexA* will continue to increase, which could confer a fitness advantage under selection from continued florfenicol usage.

## MATERIALS AND METHODS

### *Campylobacter* strains and antimicrobial susceptibility testing.

The *Campylobacter* isolates used in this study were isolated from cecal contents, carcasses, and feces of ducks and chickens in Zhejiang Province, China. All *Campylobacter* strains were grown on Mueller-Hinton (MH) agar (Sigma-Aldrich, St. Louis, MO) at 42°C under microaerobic conditions (5% O_2_, 10% CO_2_, and 85% N_2_).

Antimicrobial susceptibility testing was conducted using the standard broth dilution method according to the guidelines of the Clinical and Laboratory Standards Institute (CLSI) (20). The C. jejuni ATCC 33560 strain was used for quality control. All experiments were repeated 3 times.

### Natural transformation.

Natural transformation was conducted following the method previously described (21). Briefly, purified genomic DNA from the florfenicol-resistant C. jejuni or constructed suicide plasmids served as the donor DNA, and the naturally competent C. jejuni NCTC 11168 was used as the recipient strain. The genomic or plasmid DNA was spotted (10 μl) onto an overnight lawn of C. jejuni and incubated for 6 h at 37°C. After incubation, the lawn was harvested and plated on MH agar containing selective antimicrobial agents. Transformants were selected on MH agar plates containing florfenicol (4 μg/ml) or kanamycin (30 μg/ml). Transformation without donor DNA was used as a negative control. Transformants were confirmed using pulsed-field gel electrophoresis (PFGE) analysis, which was performed using SmaI as the restriction endonuclease following the protocol for *Campylobacter* (22). Consecutively, transformants were examined for mutations involved in florfenicol resistance.

### Construction of the *fexA* mutant.

To identify its function, the *fexA* gene from wild-type C. jejuni was knocked out by insertional mutagenesis. Primers (Table 2) *fexA*-5′F and *fexA*-5′R were used to amplify a 920-bp fragment containing the 5′ part of *fexA* and its upstream gene, while primers *fexA*-3′F and *fexA*-3′R were used to amplify the 3′ region of the *fexA* gene with 209 bp immediately downstream of the gene. The *aphA3* kanamycin resistance cassette was amplified from pMW10 using the Phusion high-fidelity DNA polymerase (New England BioLabs [NEB]). After purification, the PCR products of *fexA*-5′, *aphA3*, and *fexA*-3′ were ligated using the Gibson assembly, yielding a 3,175-bp *fexA*-5′-*aphA3-fexA*-3′ fragment. The purified PCR product was subsequently introduced into C. jejuni NCTC 11168 using natural transformation. Mutants were selected on MH agar containing 30 μg/ml of kanamycin. Insertion of the *aphA3* cassette into the *fexA* gene was confirmed by PCR analysis.

**TABLE 2:** Key primers used in this study

Primer	Sequence	PCR product size (bp)
*fexA*-5′F	GTAATGGGAATTGATTTCATTAATGTCGA	920
*fexA*-5′R	CCACAATTATGATAGAATTTACTCCACCAAATATTGGACCAG	
*aphA3*-F	AAATTCTATCATAATTGTGGTTTCAAAATCGGCT	1,215
*aphA3*-R	ACCCTAAATATTGACCAACTAAAATGTCAAAAGTTGCCACC	
*fexA*-3′F	AGTTGGTCAATATTTAGGGTGGAATGC	1,080
*fexA*-3′R	TTCGCACCAATAAAACAGTGTACG	
*fexA*-F	AGGAGAACCTGCGGTTGGATCACCTCCTTTCTAGAGCAAAAATTTATGAATCTATTGCAT	1,878
*fexA*-R	TAATAGTTGTGAGACTTATTACTTTGTACTCTAGAGAAACGATCACCAATGTTTTCATTG	
*fexA*-IF	TTTTAATGATGGTACTCTCCCT	529
*fexA*-IR	GGTAACGCGTAGTAGGCACCAA	

### Functional clone of *fexA*.

A wild-type copy of *fexA* was inserted into the chromosome between the 16S and 23S rRNAs of C. jejuni NCTC 11168 lacking the *fexA* gene, as previously described (23). The entire *fexA* gene, including its promoter region, was amplified from C. jejuni ZJB020 by PCR using primers *fexA*-F and *fexA*-R (Table 2). The pRRK plasmid containing an *aphA3* cassette in the opposite orientation to the ribosomal genes was linearized by XbaI digestion. The homologous recombination method was used to fuse the *fexA* amplicon to pRRK to obtain the pRRK-*fexA* plasmid construct. The pRRK-*fexA* suicide plasmid construct was naturally transformed into the wild-type C. jejuni NCTC 11168 strain, resulting in the insertion of a copy of *fexA* in the chromosome. Transformants were selected on MH agar plates containing 30 μg/ml of kanamycin and confirmed by PCR using primers *fexA*-IF and *fexA*-IR. The obtained transformant strain was named 11168*+fexA*.

### Genome sequencing and assembly.

Genomic DNA of all *fexA*-positive isolates was extracted using the Wizard genomic DNA purification kit (Promega, Beijing, China), following the manufacturer’s instructions. Indexed Illumina sequencing libraries were prepared using the TruSeq DNA PCR-free sample preparation kit (Illumina Inc., San Diego, CA) following the standard protocol and sequenced on the Illumina HiSeq 2500 platform according to the manufacturer’s protocols, thus producing 150-bp paired-end reads. Draft genomes were assembled using SPAdes (24) and CLC Genomics Workbench (version 8.5; CLC Bio, Aarhus, Denmark) software.

To obtain the complete genome sequence, these 9 isolates were also selected for MinION long-read sequencing. Library preparation was performed using a rapid barcoding sequencing kit (SQK-RBK004) according to the standard protocol provided by the manufacturer (Oxford Nanopore). Guppy (version 3.2.4) was used for base calling and demultiplexing of MinION long-read sequencing raw data. Consecutively, we performed a hybrid strategy of *de novo* assembly by combining Illumina short-read data and MinION long-read data using Unicycler (version 0.4.4) in order to obtain high-quality complete genomes as previously described (25, 26).

### Multilocus sequence typing.

Multilocus sequence typing (MLST) was performed by PCR and sequencing of 7 housekeeping genes (*aspA*, *glnA*, *gltA*, *glyA*, *tkt*, *pgm*, and *uncA*) (27), and analysis was performed based on the PubMLST web tool (http://pubmlst.org/campylobacter).

### Analysis of *fexA* location.

The plasmid or chromosome location of the *fexA* gene in these 9 *Campylobacter* isolates was first determined by S1 nuclease pulsed-field gel electrophoresis (PFGE) and Southern blotting as previously described (9). To this end, S1 nuclease (TaKaRa, Dalian, China) was used to digest agarose gel plugs containing cells of *fexA*-carrying isolates. Southern blotting was performed to detect the location of the *fexA* gene. The probe was amplified using the specific primers (*fexA*-S1F, 5′-CTTATCTCCCTTCGTTGGC-3′, and *fexA*-S1R, 5′- TACTGCGGCGTTATTTGC-3′) for the *fexA* gene and then labeled using a DIG High Prime I DNA labeling and detection starter kit for hybridization. Signals from the bands were visualized using a nitroblue tetrazolium–5-bromo-4-chloro-3-indolylphosphate (NBT-BCIP) color detection kit (Roche Diagnostics, Mannheim, Germany) following the recommendations of the supplier.

The location of *fexA* was further confirmed through the assembly of whole-genome sequencing, by analyzing whether it was on a plasmid or chromosome.

### Analysis of genetic context of *fexA*.

The *fexA*-carrying contigs were annotated using the RAST annotation server (28). Insertion sequences were identified by ISfinder (29). Gene prediction and annotation of the genomes were performed using the NCBI Prokaryotic Genome Annotation Pipeline. Acquired antimicrobial resistance genes were predicted using ResFinder 3.1 (https://cge.cbs.dtu.dk/services/ResFinder/). oriTfinder (http://202.120.12.134/oriTfinder/oriTfinder.html) was used to identify the origin of transfers in the genome. The BLAST Ring Image Generator (BRIG) and Easyfig software were used in the comparative analysis of plasmids.

### Accession numbers.

Complete genome sequences have been deposited in GenBank under BioProject number PRJNA605601 and GenBank accession numbers CP048756 to CP048774.

## Supplementary Material

Supplemental file 1
