# Supplementary material for: Emergence of fexA in Mediating Resistance to Florfenicols in Campylobacter
Source: Antimicrob Agents Chemother. 2020 Jun 23;64(7):e00260-20. doi: 10.1128/AAC.00260-20 (PMC7317992; doi:10.1128/AAC.00260-20)
Supplement: Supplemental file 1 [file AAC.00260-20-s0001.pdf]

Supplementary figures

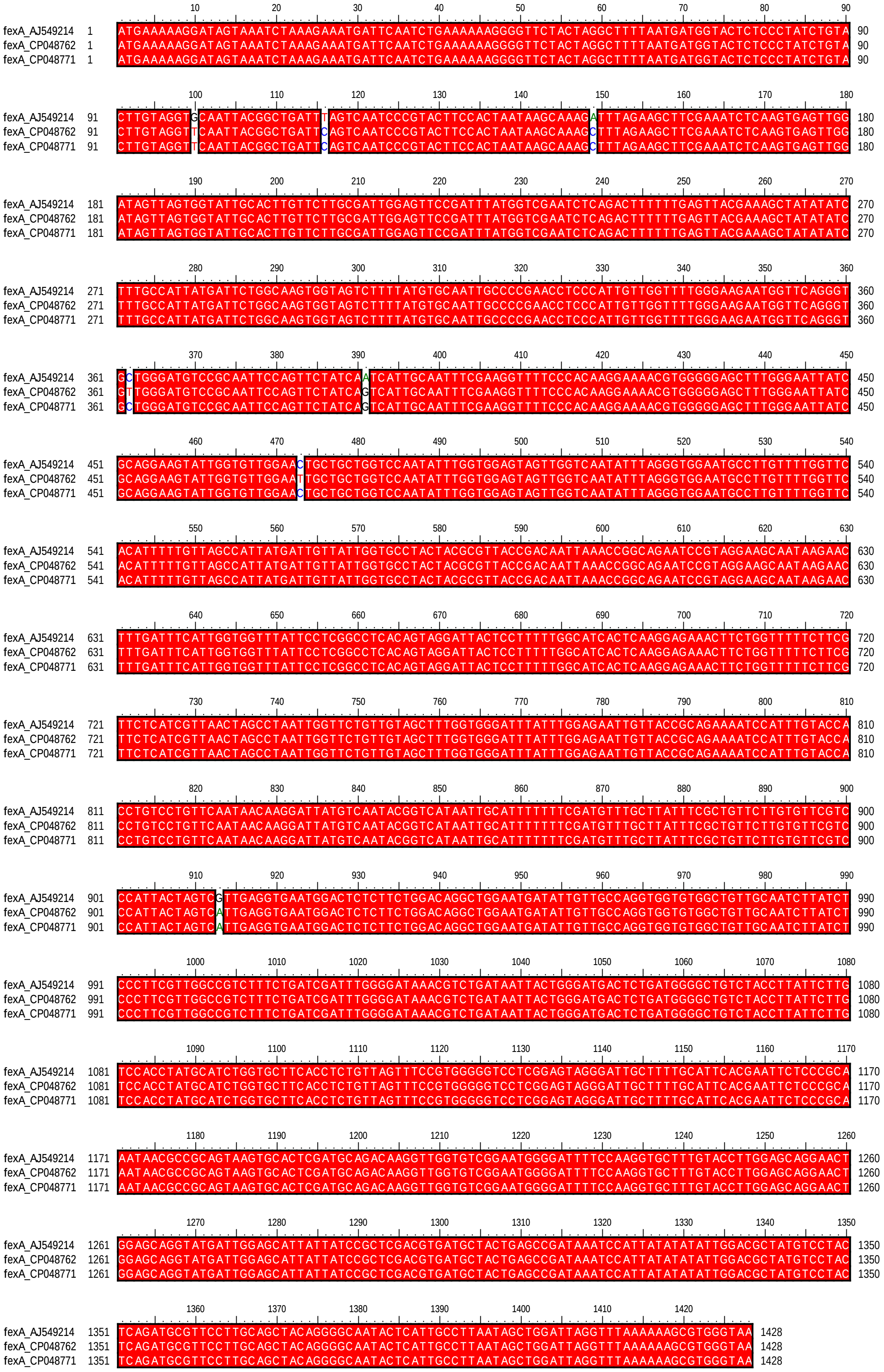

Figure S1. Comparison of nucleic acid sequences of *fexA* gene.

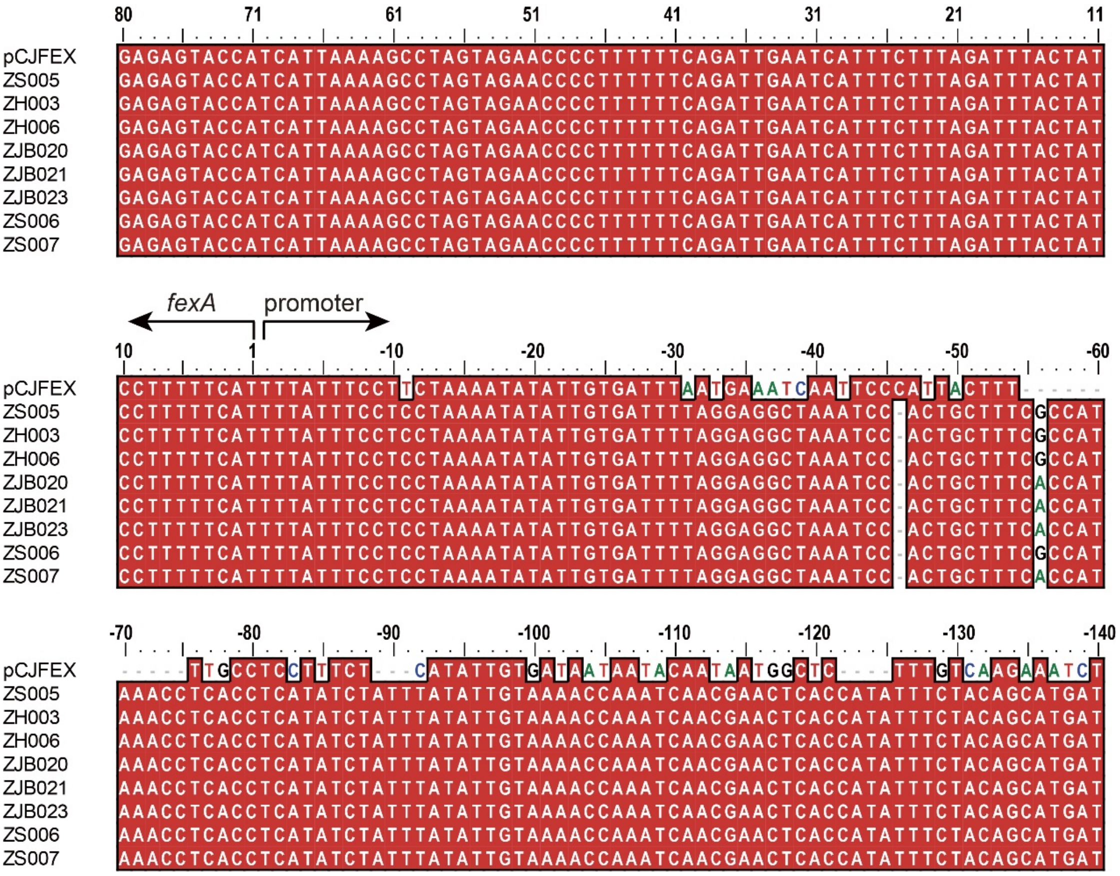

Figure S2. Comparison of promoter regions of *fexA* gene.

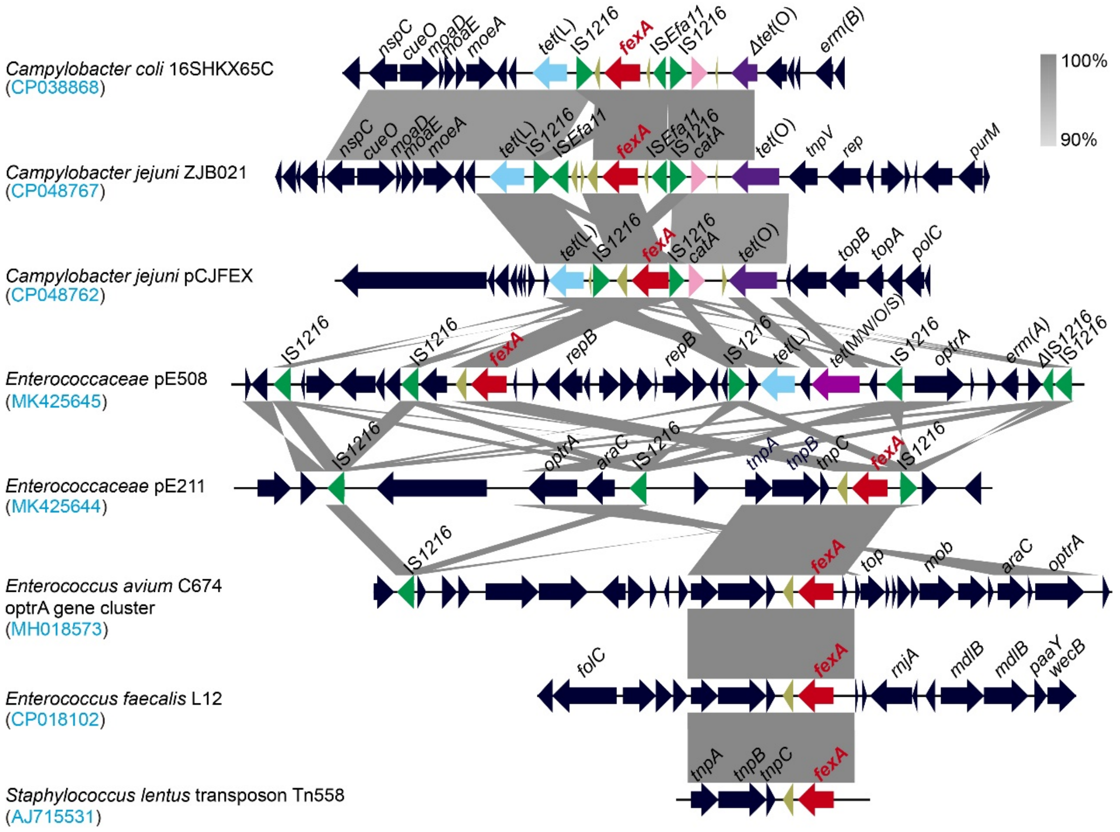

Figure S3. Genetic environment contents of *fexA* gene in staphylococci, enterococci and *Campylobacter*.
